# Controlling the elasticity of polyacrylonitrile fibers *via* ionic liquids containing cyano-based anions†

**DOI:** 10.1039/d2ra00858k

**Published:** 2022-03-18

**Authors:** Zongyu Wang, Huimin Luo, Halie J. Martin, Tao Wang, Yifan Sun, Mark A. Arnould, Bishnu P. Thapaliya, Sheng Dai

**Affiliations:** Chemical Sciences Division, Oak Ridge National Laboratory Oak Ridge Tennessee 37831 USA dais@ornl.gov; Manufacturing Science Division, Oak Ridge National Laboratory Oak Ridge Tennessee 37831 USA luoh@ornl.gov; Department of Chemistry, The University of Tennessee Knoxville Tennessee 37996 USA; Center for Nanophase Materials Sciences, Oak Ridge National Laboratory Oak Ridge Tennessee 37831 USA

## Abstract

As the predominant precursor for high-performance carbon fiber manufacturing, the fabrication of polyacrylonitrile (PAN)-based composite fibers attracts great interest. Ionic liquids (ILs) have recently been investigated for melt-spinning of ultrafine PAN fibers. The plasticizing properties of ILs are significantly affected by the structure of ILs and can be influenced by electronegativity, steric effects, *etc.* Herein, we report a facile strategy to control the elasticity of the PAN/ILs fibers by tuning the anion structure of ILs. Particularly, the ILs containing nitrile-rich groups exhibited enhanced plasticizing effect and nucleating ability on dissolving PAN components, achieving highly stretchable PAN/ILs fibers.

## Abstract 2

Highly stretchable PAN/ILs fibers were fabricated through melt-spinning with ionic liquids containing cyano-based anions.

## Graphical abstract

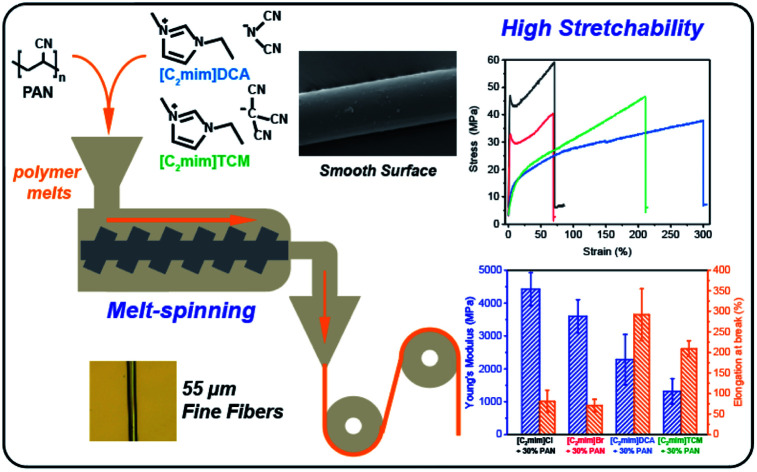

The unique properties of lightweight, excellent chemical resistance, and high strength make carbon fiber reinforced composites emerging materials for producing advanced functional composites for the aircraft, automobile, wind power, and sports equipment industries.^1,2^ Despite the continuous market growth, the widespread use of carbon fibers is limited to high-end products primarily due to their tedious manufacturing steps and extensive capital investment. A substantial fraction of the expense results from the energy input needed to convert the fiber precursor into carbon fibers.^3–6^ On the other hand, the mechanical performance (elastic modulus and tensile strength) of carbon fibers directly relies on the properties of the precursor.^7^ Polyacrylonitrile (PAN) is the preferred precursor employed for the manufacture of carbon fibers, leading the marketplace with a share of around 90%. PAN endows fibers with extraordinary mechanical characteristics and high production outputs.^4^ Meanwhile, carbon fibers derived from precursor fibers other than PAN or its derivatives exhibit considerably inferior mechanical performance.^8^ Thus, it is crucial to enhance the efficiency of the fiber spinning of PAN and its derivatives to cut down the total expense while keeping the merits of PAN as the precursor for carbon fibers.^9,10^

Generally, PAN-based fibers for high-strength carbon fibers manufacturing are spun through a solution spinning procedure, usually referred to as wet-spinning. Due to the high polarity of the nitrile group, solution spinning requires the use of highly polar solvents including dimethyl sulfoxide (DMSO), *N*,*N*-dimethylmethanamide (DMF), *N*-methyl-2-pyrrolidone (NMP), which accounts for the major production expense of PAN fibers.^6,11–16^ As an alternative to solving this dilemma, the melt-spinning process has been developed as an efficient method for fabricating polymer nanofibers. In recent years, various polymers have been successfully melt-spun into ultrafine or nanometer fibers in melt forms.^17–20^ This technique is particularly attractive for fabricating low-cost carbon fibers as it has a high manufacturing speed and eliminates the additional cost of solvent recycling.^21–25^

However, direct melt-spinning of PAN precursor is challenging largely owing to its high melting point (∼300 °C), at which temperature PAN begins to undergo thermal decomposition. External plasticization is frequently used to decouple the strong dipole–dipole interactions among the nitrile groups, which results in a decreased softening point below the PAN decomposition temperature. Examples of commonly used plasticizers include water, acetonitrile, dimethylformamide, alcohol, or co-solvents.^24^ Ionic liquids (ILs), as potential “green solvents”, are ideal candidates for fiber impregnation not only because they possess negligible volatility, excellent thermal and chemical stability, and low toxicity,^26,27^ but also is attributed to their brilliant capacity of dissolving natural and synthetic polymers,^28^ such as cellulose,^29,30^ chitosan,^31^ keratin,^32,33^ silk,^34^ and PAN^34^ based on hydrogen bond theory.^35–37^ The ILs are called the “designer solvents”,^38,39^ as various properties and novel functionalities can be introduced to the material by accommodating a broad scope of component ions. Besides, many recent works have demonstrated the design of new series of ionic liquids with a low-cost synthesis route.^40–44^ Although the major explorations of ILs have been inclined to focus on the development of the cationic structure modifications, the influence of cation chain length plays no major role during dissolution.^45^ Meanwhile, the plasticizing properties of ILs are profoundly influenced by substituents of anions and may be systematically affected by the size, electronegativity, steric effects, *etc.*^46–48^

In this contribution, highly stretchable fibers composed of PAN and ILs were fabricated through the melt-spinning method. Four ILs with the same cation, 1-ethyl-3-methylimidazolium cation ([C_2_mim]^+^), but different counter anions, from small halogen hydrogen-bond acceptors (Cl^−^/Br^−^) to larger polycyano anions (dicyanamide [N(CN)_2_]^−^) and triacyanomethanide ([C(CN)_3_]^−^), were incorporated with PAN to fabricate fibers (Fig. 1). Compared to the halides, the polycyano anions possess a higher electron-withdrawing ability, are expected to have stronger interaction with the PAN polymers, which can potentially lead to superior mechanical performance in the PAN/IL fiber composites. The main reason to select 1-ethyl-3-methylimidazolium cation is that the ILs with this cation have lower melting points and are less viscous than longer chain imidazolium ILs. In addition, the four ILs used in our papers are commercially available. The thermal analyses including thermogravimetric analysis (TGA) and differential scanning calorimetry (DSC) were used to characterize the decomposition behavior, thermal stability as well as carbon yields of the PAN/ILs fibers. The morphological study and X-ray diffraction (XRD) were applied to determine the plasticizing effect and nucleating ability of each IL on the PAN composite fibers. The mechanical properties, particularly the elastic modulus and elongation at break, were investigated by tensile tests using an Instron 5943 universal testing machine. Besides, the regenerated PAN fibers made by removing ILs with deionized water were prepared for comparison.

**Fig. 1:**
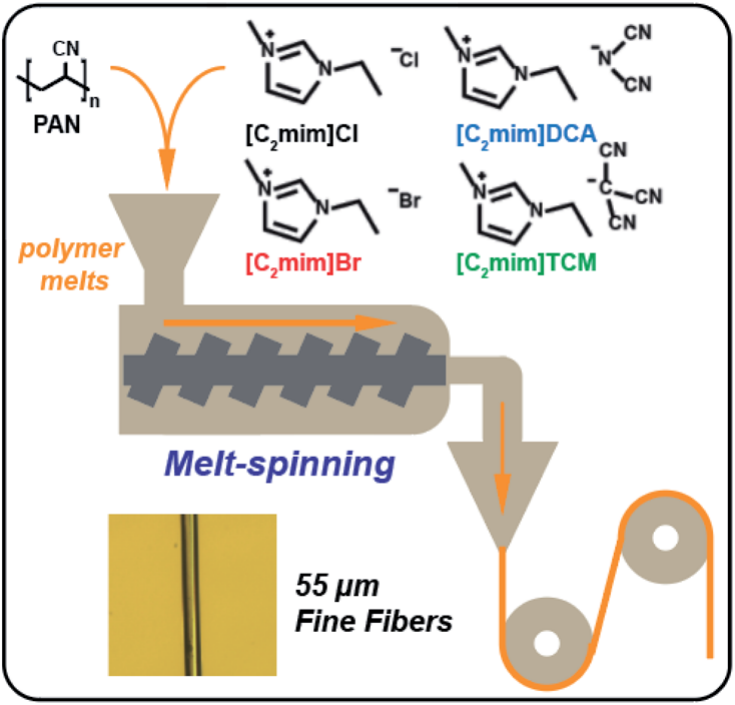
Schematic illustration of the preparation of PAN/ILs composite fibers.

Dry fine PAN powders (*M*_n_ = 52 290) were first mixed with IL, then heated to ∼160 °C under nitrogen to prepare homogenous PAN/ILs melts with a PAN : ILs = 30 : 70 weight ratio. The composite melts were then melt-spun to form uniform fibers (∼55 μm diameter) using a melt extruder following the procedures from the previous report.^12^ One of the most important applications of PAN fibers is to be employed as the precursors for high-performance carbon fiber manufacturing. PAN fibers are converted to carbon fibers *via* oxidative cyclization/stabilization followed by carbonization. The cyclization/stabilization step converts –C≡N groups to cyclic –C=N– and –C–N– groups and is typically carried out in the air in the temperature range of 200–300 °C. The carbonization temperature generally ranges from 400 to 800 °C.^49–51^ The performance of the resultant carbon fibers is thus strongly associated with the cyclization process,^52,53^ and the kinetics of cyclization can be modified by tuning the incorporated ingredients with PAN, such as carbon nanotubes, lignin, or cellulose nanocrystals,^54–56^ which might facilitate the primary cyclization reaction and consequently increase the carbon yield.

Before investigating the thermal behaviors of the PAN/ILs fibers, the thermal analysis of the pure ILs, PAN powders was conducted with TGA (scan ranges up to 800 °C under N_2_ atmosphere) and DSC (scan ranges from −50 °C to 180 °C). As shown in the TGA plots (Fig. S3a†), the ILs with halide anions exhibited 0 wt% carbon yield, and the nitrile-rich IL [C_2_mim]TCM gave a similar carbon yield as PAN powders (∼30 wt%, Table S1†), while [C_2_mim]DCA's ∼11 wt%. The DSC curves of the pure ILs and PAN powder were included in Fig. S2c and S3c,† from where the crystallization and melting points of [C_2_mim]Cl, [C_2_mim]Br, and [C_2_mim]DCA were obtained (Table S1†). However, no peaks were observed in [C_2_mim]TCM and PAN within the same scanning range. The polycyano anions: dicyanamide and triacyanomethanide are important because of their versatility in application to broad fields of materials science.^57^ Due to their high contents of nitrile (–CN) moieties, higher carbon yields are expected in the corresponding hybrid fibers. The thermal decomposition of the four PAN/ILs fibers is shown in Fig. 2a, and surprisingly, high carbon yields were observed in all of the fiber samples, as PAN/[C_2_mim]Cl: 50 wt%, PAN/[C_2_mim]Br: 46 wt%, PAN/[C_2_mim]DCA: 48 wt% and PAN/[C_2_mim]TCM: 53 wt%. For comparison, TGA plots of the washed PAN fibers' were included in Fig. S6a,† which gave ∼43 wt% carbon yields. This indicates that a strong interaction was generated between the PAN and ILs during the extrusion processing, and besides the PAN, ILs also acted as the precursor and contributed to the high carbon yields. The strong interaction between PAN and ILs was also confirmed by the DSC curves of the composite fibers, Fig. 2c, as the melting/crystallization points of ILs can no longer be observed in the heat flow plots of PAN/ILs fibers.

**Fig. 2:**
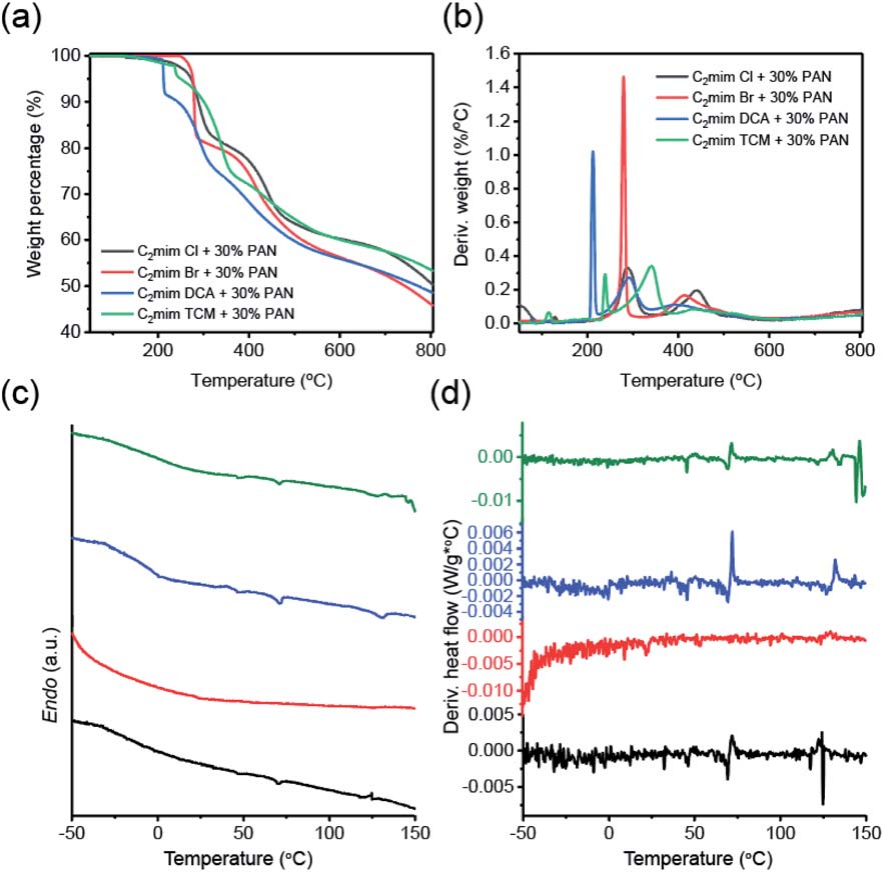
(a) TGA curves of PAN/ILs fibers, (b) the plots of derivative weight *vs.* temperature of PAN/ILs fibers, (c) DSC curves of PAN/ILs fibers (d) the plots of derivative heat flow *vs.* temperature of PAN/ILs fibers, black line: [C_2_mim]Cl; red line: [C_2_mim]Br; blue line: [C_2_mim]DCA; olive line: [C_2_mim]TCM.

The morphological features of PAN/ILs hybrid fibers were first examined by optical microscopy (Fig. S7†) and scanning electron microscopy (SEM). The structures of the prepared PAN/[C_2_mim]Cl and PAN/[C_2_mim]TCM fibers before and after the PAN regeneration procedure are illustrated in Fig. 3 schematically. All samples exhibited a smooth surface morphology with an average diameter size of around 50 μm, indicating excellent compatibility of ILs with PAN. No significant difference was observed between the PAN/ILs fiber and the regenerated PAN fibers after the ILs' removal procedure by deionized water.

**Fig. 3:**
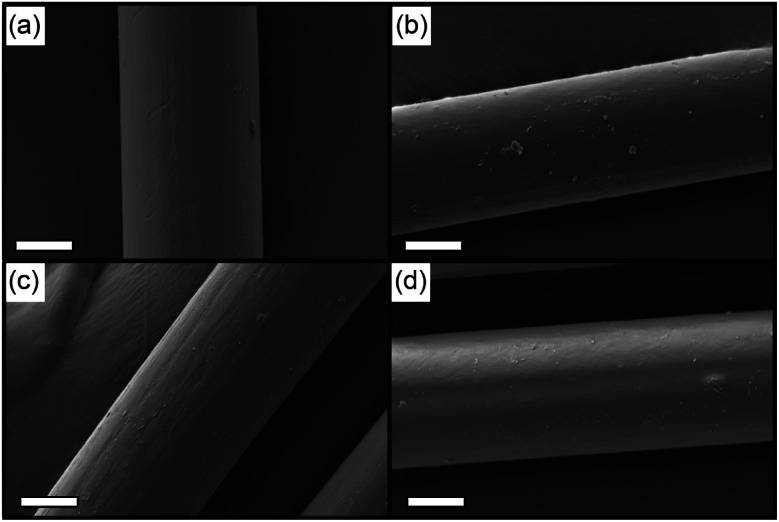
SEM images of (a) PAN/[C_2_mim]Cl fibers, (b) PAN/[C_2_mim]TCM fibers, (c) washed PAN/[C_2_mim]Cl fibers, (d) washed PAN/[C_2_mim]TCM fibers, scale bars: 20 μm.

As there are no clear differences obtained from thermal and morphological studies between the halide anions and polycyano anions ILs-based composite fibers, XRD was used to understand the interactions of PAN with ILs, which provides further insights into the investigation and evaluation of the plasticizing effect of the imidazolium-based ILs with nitrile-rich counter anions. The fibers were chopped into small pieces for the measurements and XRD patterns of four samples were shown in Fig. 4. The peak at 17.5° is the characteristic peak of PAN. The XRD results indicate that PAN/ILs with polycyano anions exhibited substantially higher crystallinity and slightly larger crystal size than the PAN/ILs with halide anions, as the higher intensity and sharper peaks shown in the patterns. Compared to ILs with halide anions, the multiple cyano moieties with high electron-withdrawing ability in polycyano anions are anticipated to induce more favorable interaction with PAN chains, which could benefit the dissolution and nucleation process. These enhanced plasticizing and nucleating effects result in higher crystallinity and slightly larger PAN crystal size in PAN/[C_2_mim]DCA and PAN/[C_2_mim]TCM fibers, as evidenced by the emergence of the sharper diffraction peaks. Our XRD results suggested that the PAN/ILs with polycyano anions fibers are expected to present better mechanical performance.

**Fig. 4:**
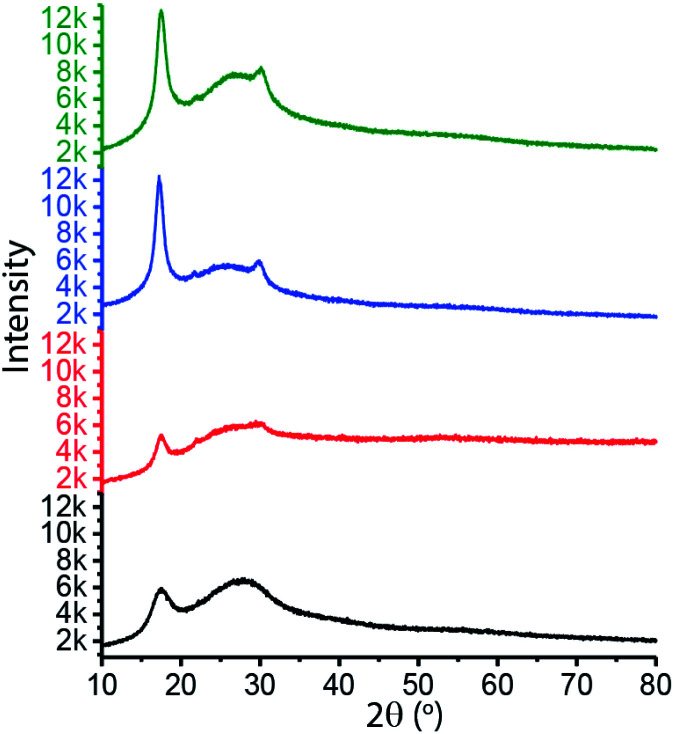
XRD comparison of four PAN-IL fibers: PAN/[C_2_mim]Cl fibers (black line), PAN/[C_2_mim]Br fibers (red line), PAN/[C_2_mim]DCA fibers (blue line), PAN/[C_2_mim]TCM fibers (olive line).

The ability to preserve morphological integrity upon large deformation is critical to ensure a bulk material maintains reliable operation.^58^ Compared with film-based devices, the fibrous ones are advantageous considering the convenience for packaging and thus particularly suitable for undersea applications.^59–62^ Desired mechanical performance is also necessary to afford the materials to be employed in harsh environments, including transport, working, handling, and installation. However, it is challenging to achieve high elasticity in three-dimensional fibrous devices. Mechanical property evaluations of PAN/ILs fibers were carried out using an Instron 5943 universal testing machine. Twenty-five specimens were characterized for each fiber type (Fig. S4†) and the representative strain–stress curves were included in Fig. 5a. The determination of Young's modulus from the stress–strain curves is limited to the low-strain regime below 5% strain. The strain–stress curves of PAN/[C_2_mim]Cl and PAN/[C_2_mim]Br fibers exhibited typical ductile material features, in where the clear yield points (∼1.5% strain) and strain hardening were observed. On the other hand, PAN/[C_2_mim]DCA and PAN/[C_2_mim]TCM fibers show more elastomer-like features in the strain–stress curves with significantly higher elongation at break values, reaching 300% and 210%, respectively (Fig. 5b). A plausible explanation for this difference between PAN/ILs with halide anions and PAN/ILs with polycyano anions is that as the PAN and ILs exhibit excellent compatibility at PAN : ILs = 30 : 70 (wt%) ratio, the incorporation of ILs results in a fine PAN crystal distribution. The introduction of ILs with nitrile-rich groups showed a better plasticizing and nucleating ability than those ILs with halides anions. With high crystal nucleating density and fine crystal size, the mechanical properties of PAN/[C_2_mim]DCA and PAN/[C_2_mim]TCM fibers achieved a satisfactory enhancement. The higher stretchability exhibited in PAN/[C_2_mim]DCA fibers is probably due to the stronger coordination ability of [N(CN)_2_]^−^ than [C(CN)_3_]^−^.^57^ Besides, the performance of the regenerated PAN fibers was characterized for comparison (Fig. S5 and Table S2†). A higher Young's modulus and lower elongation at break values were obtained, which indicated that in a PAN/ILs fiber, the PAN provides mechanical robustness, while the IL offers sufficient elasticity.

**Fig. 5:**
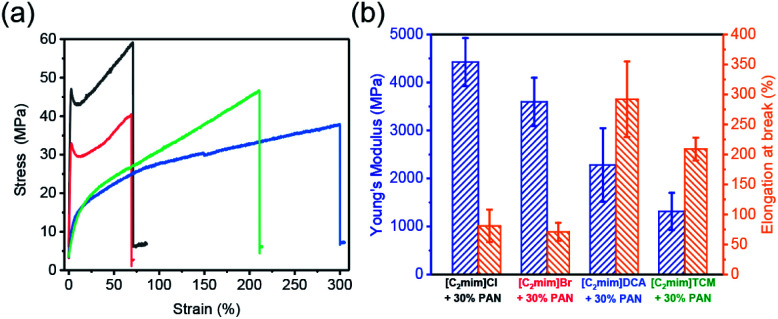
(a) Representative strain–stress curves of PAN/IL fibers, black: [C_2_mim]Cl/PAN fibers, red: [C_2_mim]Br/PAN fibers, blue: [C_2_mim]DCA/PAN fibers, olive: [C_2_mim]TCM/PAN fibers, (b) Young's modulus and elongation at break of PAN/ILs fibers.

In this work, we present a robust methodology for fabricating highly stretchable PAN/ILs fibers *via* the melt-spinning technique using ILs with polycyano anions. The selection of different ILs could profoundly affect the properties of the PAN composite fibers. ILs containing strong electronegativity halide anions such as [C_2_mim]Cl/[C_2_mim]Br, and ILs with polycyano anions ([C_2_mim]DCA/[C_2_mim]TCM) which have high electron-withdrawing ability were screened to investigate their plasticizing effect on the PAN fibers. With 30 wt% PAN loading in the composite fibers, all four ILs exhibited excellent compatibility with PAN. The PAN/ILs fibers also showed high carbon yields, which even exceed that of the fresh and regenerated PAN fibers. This indicated a strong interaction was induced between the PAN chains and the ILs through the extruding procedure. Besides the PAN, the incorporated ILs were also attributed to the high carbon yields as the carbon precursors. In the tensile tests, in dramatic contrast to the brittle nature of PAN fibers, the PAN/ILs fiber samples with 70 wt% [C_2_mim]DCA/[C_2_mim]TCM ILs exhibited outstanding mechanical properties for large deformations (300%/210%). Compared to the PAN/[C_2_mim]Cl and PAN/[C_2_mim]Br fibers, which showed typical ductile material features, more elastomer-like featured strain–stress curves were observed in PAN/[C_2_mim]DCA and PAN/[C_2_mim]TCM fibers. These significantly enhanced mechanical performances can be attributed to the better plasticizing and nucleating ability of nitrile-rich ILs, which is supported by the results of higher PAN crystallinity and slightly larger crystal size from XRD patterns. This work demonstrated that rational incorporation with appropriate functional anions would not only extend the ongoing exploration of novel and versatile ILs but also help understand the correlation between the molecular structures of component ions and composite properties. The developed PAN/ILs fibers, which exhibit tunable and excellent mechanical properties, assure the material with promising prospects for extensive applications in various fields.

## Conflicts of interest

The authors declare that they have no known competing financial interests or personal relationships that could have appeared to influence the work reported in this paper.

## Supplementary Material

RA-012-D2RA00858K-s001
