# Supplementary material for: Controlling the elasticity of polyacrylonitrile fibers via ionic liquids containing cyano-based anions
Source: RSC Adv. 2022 Mar 18;12(14):8656–60. doi: 10.1039/d2ra00858k (PMC8984951; doi:10.1039/d2ra00858k)
Supplement: RA-012-D2RA00858K-s001 [file RA-012-D2RA00858K-s001.pdf]

Electronic Supplementary Material (ESI) for RSC Advances.
This journal is © The Royal Society of Chemistry 2022

Supporting Information

Controlling Elasticity of Polyacrylonitrile Fibers *via* Ionic Liquids Containing Cyano-based Anions

Zongyu Wang[a], Huimin Luo[b,*], Halie J. Martin[a], Tao Wang[a], Yifan Sun[a], Mark A. Arnould[d], Bishnu P. Thapaliya[a], Sheng Dai[a,c,*]

[a] Chemical Sciences Division, Oak Ridge National Laboratory, Oak Ridge, Tennessee 37831, United States

[b] Manufacturing Science Division, Oak Ridge National Laboratory, Oak Ridge, Tennessee 37831, United States

[c] Department of Chemistry, The University of Tennessee, Knoxville, Tennessee 37996, United States

[d] Center for Nanophase Materials Sciences, Oak Ridge National Laboratory, Oak Ridge, Tennessee 37831, United States

Corresponding Author

* E-mail: luoh@ornl.gov
* E-mail: dais@ornl.gov

Experimental Section

Materials.

Polyacrylonitrile (PAN) powder (M_n = 52,290) was obtained from Carbon and Composites Group at Oak Ridge National Laboratory. 1-ethyl-3-methylimidazolium chloride ([C_2mim]Cl, 98%, Sigma-Aldrich), 1-ethyl-3-methylimidazolium bromide ([C_2mim]Br, 98.5%, Sigma-Aldrich), 1-ethyl-3-methylimidazolium dicyanamide ([C_2mim]DCA, 98.5%, Sigma-Aldrich), 1-ethyl-3-methylimidazolium triacyanomethanide ([C_2mim]TCM, 98%, TCI Chemicals) were used as received without further purification, and their structures can be found in **Fig. 1**.

Procedures.

Preparation and Fabrication of PAN/ILs Fibers through Melt-Spinning

Dry white PAN powder was mixed with the ionic liquid in a 20 mL glass vial. The PAN/ILs mixture (with 30/70 PAN/ILs weight ratio) was purged with argon for 1 minute and heated to 160 °C until a homogeneous melt was formed.

Melt-spinning experiments were performed with an Atlas Laboratory Melt Extruder and take-up system (TUS) following the previous work.[1] The rotor temperature was set to 150 °C, the header temperature was set to 160 °C, and the rotational speed was set to 90 rpm. The polymer thread (extrudate) was wrapped around the take-up spool with tweezers when the polymer composite melts were extruded from the orifice. Once the thread was wrapped around the spool, the take-up speed was maintained at 60 feet/minute.

Ionic liquid Recovery

The ionic liquids in the polymer composite were removed by contacting the as-spun fibers with deionized water for ~24 h and then dried under 100 °C for 24 h.

Characterization.

Size Exclusion Chromatography (SEC). Number-average molecular weights (M_n) and molecular weight distributions (M_w/M_n) of PAN powders were determined by SEC in DMF as an eluent at 35 °C.

Thermogravimetric Analysis (TGA). TGA with TA Instruments 2950 was used to investigate the thermal decomposition behaviors of ionic liquids, PAN powders, and the PAN/ILs polymer composite fibers in the N_2 atmosphere. The data were analyzed with TA Universal Analysis. The heating procedure involved four steps: (1) jump to 120 ºC; (2) hold at 120 ºC for 10 min; (3) ramp up at a rate of 20 ºC/min to 800 ºC; (4) hold for 2 min. The TGA plots were normalized to the total weight after holding at 120 ºC.

Differential scanning calorimetry (DSC). The glass transition temperature (T_g), crystallization temperature (T_c) and melting temperature (T_m) of ionic liquids, PAN powders, and the PAN/ILs fibers were measured by differential scanning calorimetry (DSC) with TA Instrument QA-100. The same procedure was run three times, each involving the following steps: (1) Equilibrate at 25.00 °C, (2) Isothermal for 1.00 min, (3) Ramp 20.00 °C/min to -60.00 °C, (4) Isothermal for 1.00 min, (5) Ramp 20.00 °C/min to 150.00 °C, (6) Isothermal for 1.00 min, (7) Ramp 20.00 °C/min to -60.00 °C, (8) Isothermal for 1.00 min, (9) Ramp 20.00 °C/min to 150.00 °C, (10) Isothermal for 1.00 min, (11) Ramp 20.00 °C/min to -60.00 °C, (12) Isothermal for 1.00 min, (13) Ramp 20.00 °C/min to 180.00 °C, (14) Isothermal for 1.00 min, (15) Jump to 25.00 °C. The DSC data were analyzed with a TA Universal Analysis instrument, and T_g, T_c, and T_m were directly acquired.

Scanning and Electron Microscopy (SEM). SEM was carried out using a ZEISS AURIGA CROSSBEAM FIB scanning electron microscope to survey the surface morphologies of the PAN/ILs fibers before and after the removal of ionic liquids by deionized water.

Mechanical Analysis. The as-spun and washed PAN/ILs fibers were tested in the tensile mode by using an Instron 5943 universal testing machine. The fibers were fixed on paper mounts with a 25.4 mm gauge length and 10 mm/min speed at room temperature. 10 tests were performed for each PAN/ILs fiber to determine the average elastic modulus, tensile strength, and elongation at break values.

X-ray diffraction (XRD**).** XRD analysis using Cu *Kα* (λ = 0.1542 nm) of the PAN/ILs fibers was conducted using a PANalytical Empyrean X-ray diffractometer, operated at 45 kV and 40 mA (scanned at 0.026° per step).

Optical Microscope. The photographs of PAN/IL fibers were obtained using a Nikon Eclipse TE2000 Inverted Microscope.

Supporting data:

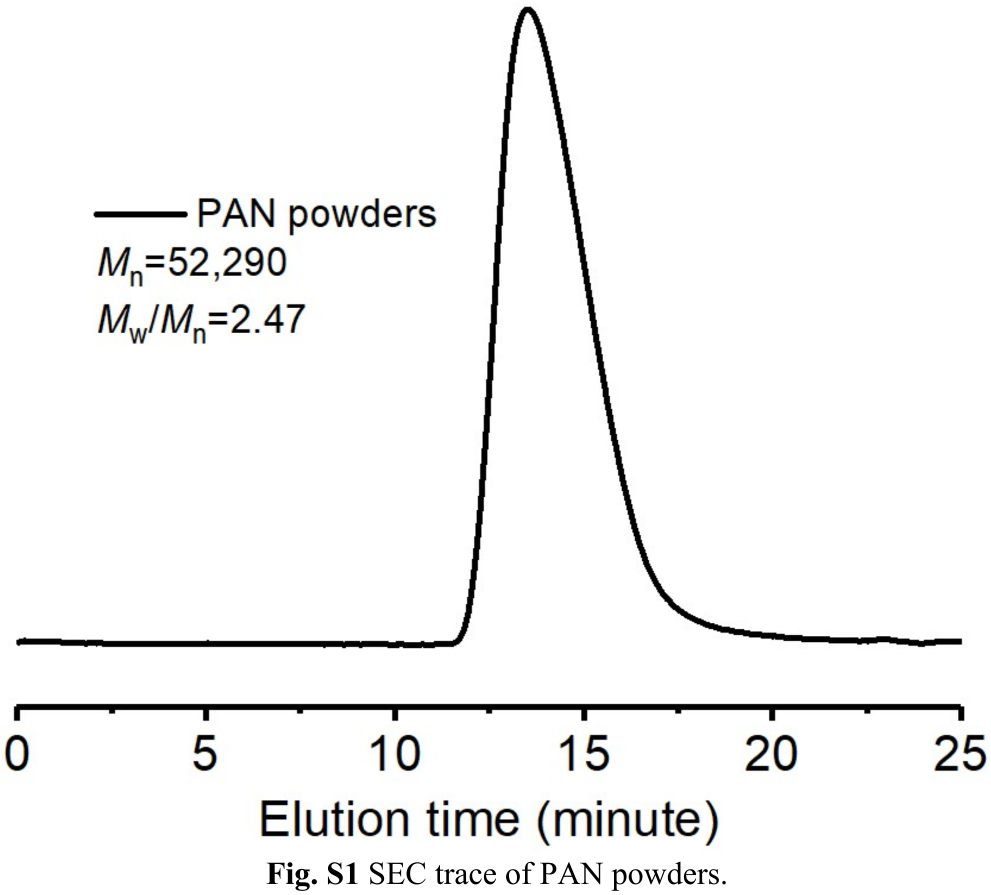

Fig. S1 SEC trace of PAN powders.

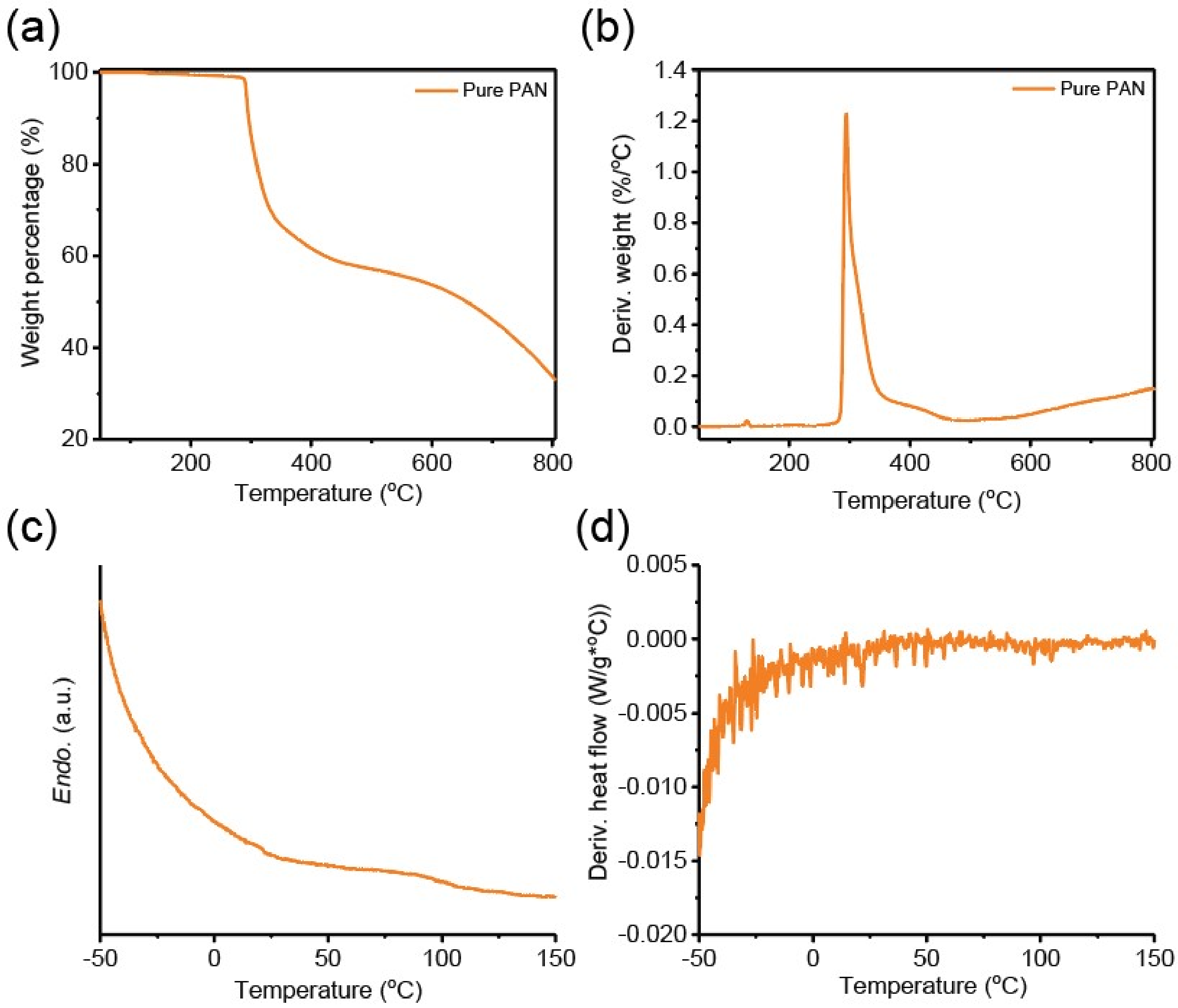

Fig. S2 (a) TGA curve of pure PAN, (b) The plot of derivative weight vs. temperature of pure PAN, (c) DSC curve of pure PAN, (d) The plot of derivative heat flow vs. temperature of pure PAN.

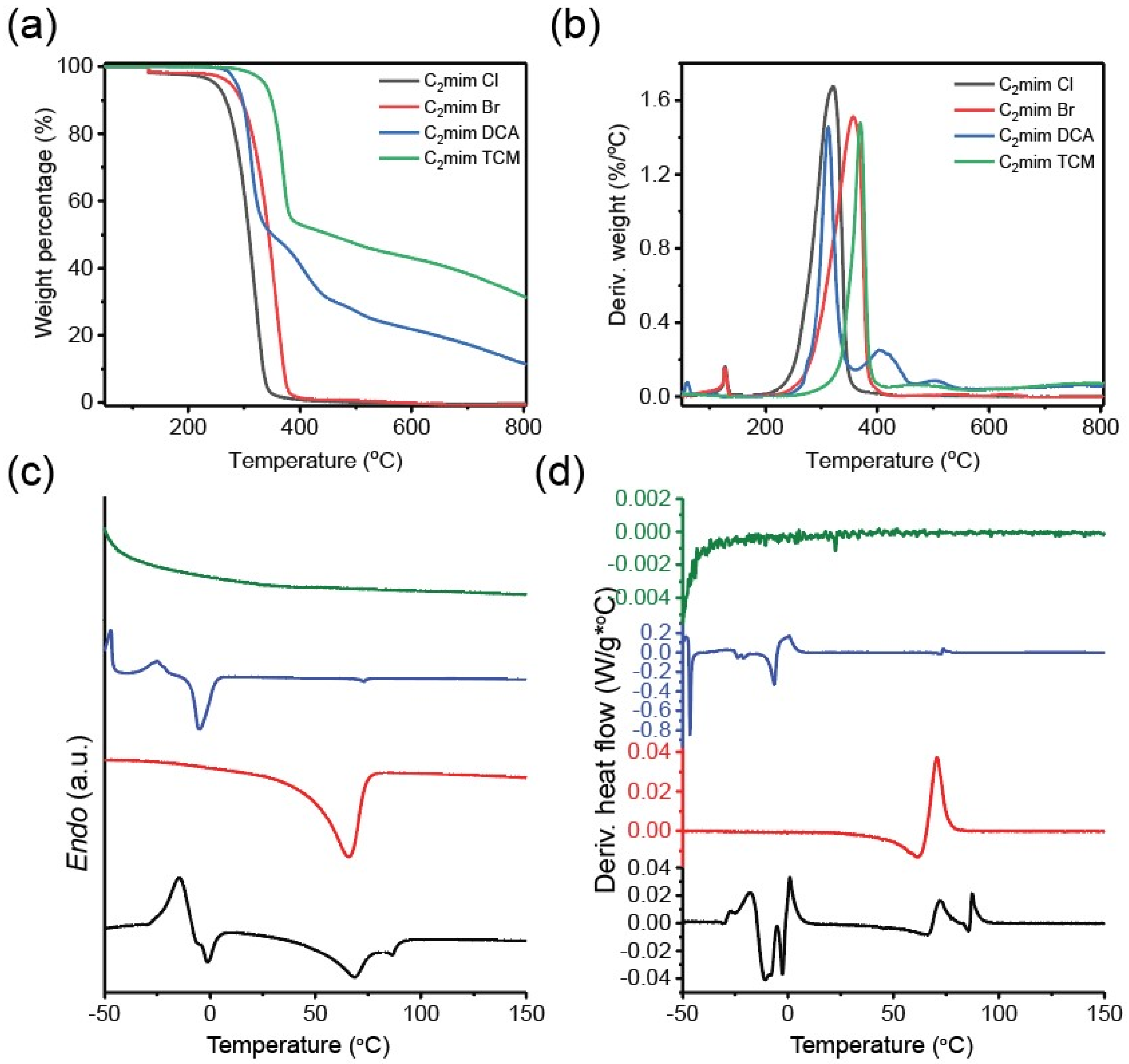

Fig. S3 (a) TGA curves of pure ILs, (b) The plots of derivative weight vs. temperature of pure ILs, (c) DSC curves of pure ILs, (d) The plots of derivative heat flow vs. temperature of pure ILs, black: [C_2mim]Cl, red: [C_2mim]Br, blue: [C_2mim]DCA, olive: [C_2mim]TCM.

Table S1. Thermal properties of pure PILs and PAN

Entry	T_d^a	Carbon yield (%) at 800 °C[a]	T_c^b	T_m^b
[C_2mim]Cl	320	0	-14.5	69.5
[C_2mim]Br	360	0	-	65.5
[C_2mim]DCA	315	11.5	-47.5	-5.5
[C_2mim]TCM	370	31.5	-	-
Pure PAN	295	33.1	-	-

[a] The decomposition temperature (T^d) and the carbon yield are determined by TGA. [b] The crystallization temperature and the melting temperature are determined by DSC.

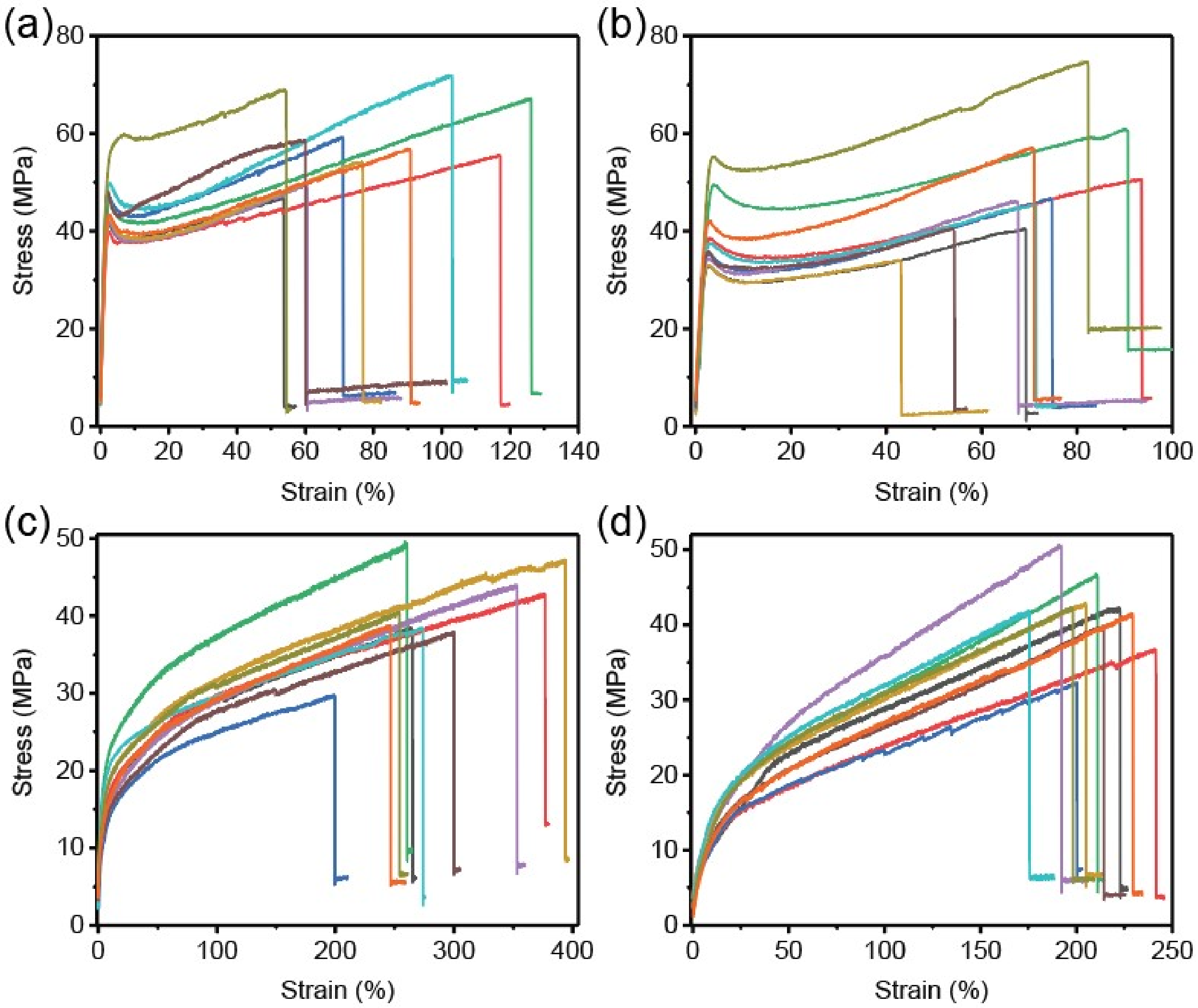

Fig. S4 Strain-stress curves of PAN/ILs fibers: (a) PAN/[C_2mim]Cl, (b) PAN/[C_2mim]Br, (c) PAN/[C_2mim]DCA, (d) PAN/[C_2mim]TCM.

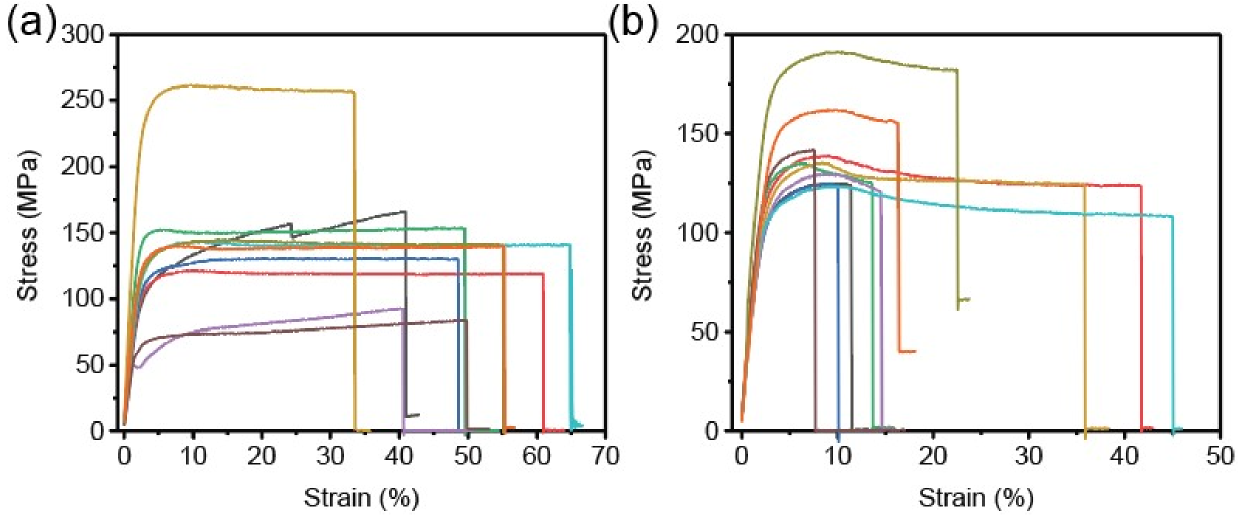

Fig. S5 Strain-stress curves of washed PAN/ILs fibers: (a) washed PAN/[C_2mim]Cl fibers, (b) washed PAN/[C_2mim]TCM fibers.

Table S2. Mechanical properties of PAN/ILs fibers

Entry	Young's modulus (MPa)[a]	Elongation at break (%)[a]
PAN/[C_2mim]Cl	4425 ± 502	81 ± 27
PAN/[C_2mim]Br	3596 ± 503	71 ± 15
PAN/[C_2mim]DCA	2280 ± 765	292 ± 63
PAN/[C_2mim]TCM	1314 ± 387	209 ± 19
Washed PAN/[C_2mim]Cl	8943 ± 4487	49 ± 10
Washed PAN/[C_2mim]TCM	8188 ± 2180	22 ± 14

[a] Determined by Instron.

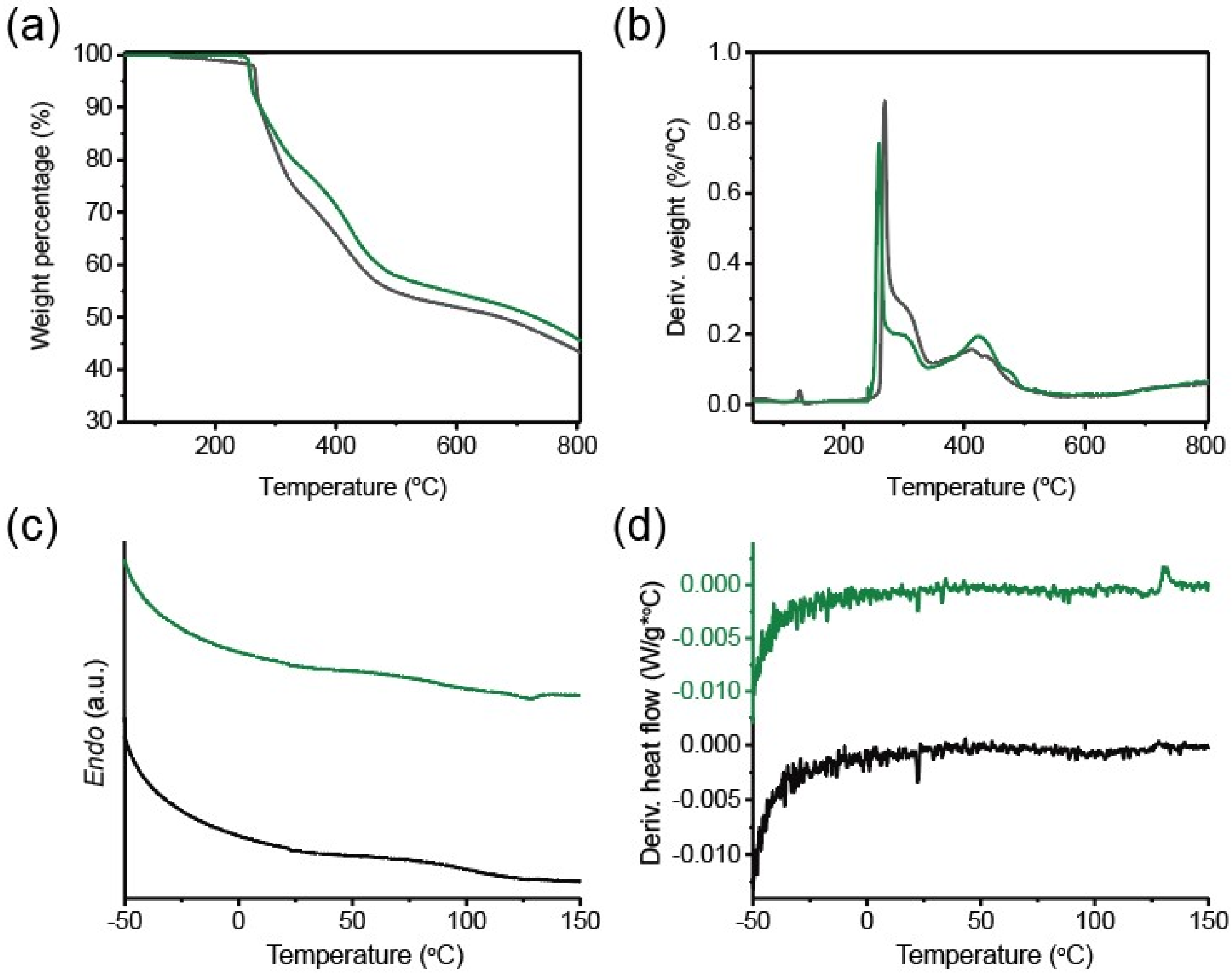

Fig. S6 (a) TGA curves of washed PAN/ILs fibers, (b) The plots of derivative weight vs. temperature of washed PAN/ILs fibers, (c) DSC curves of washed PAN/ILs fibers, (d) The plots of derivative heat flow vs. temperature of washed PAN/ILs fibers, black: PAN/[C_2mim]Cl, olive: PAN/[C_2mim]TCM.

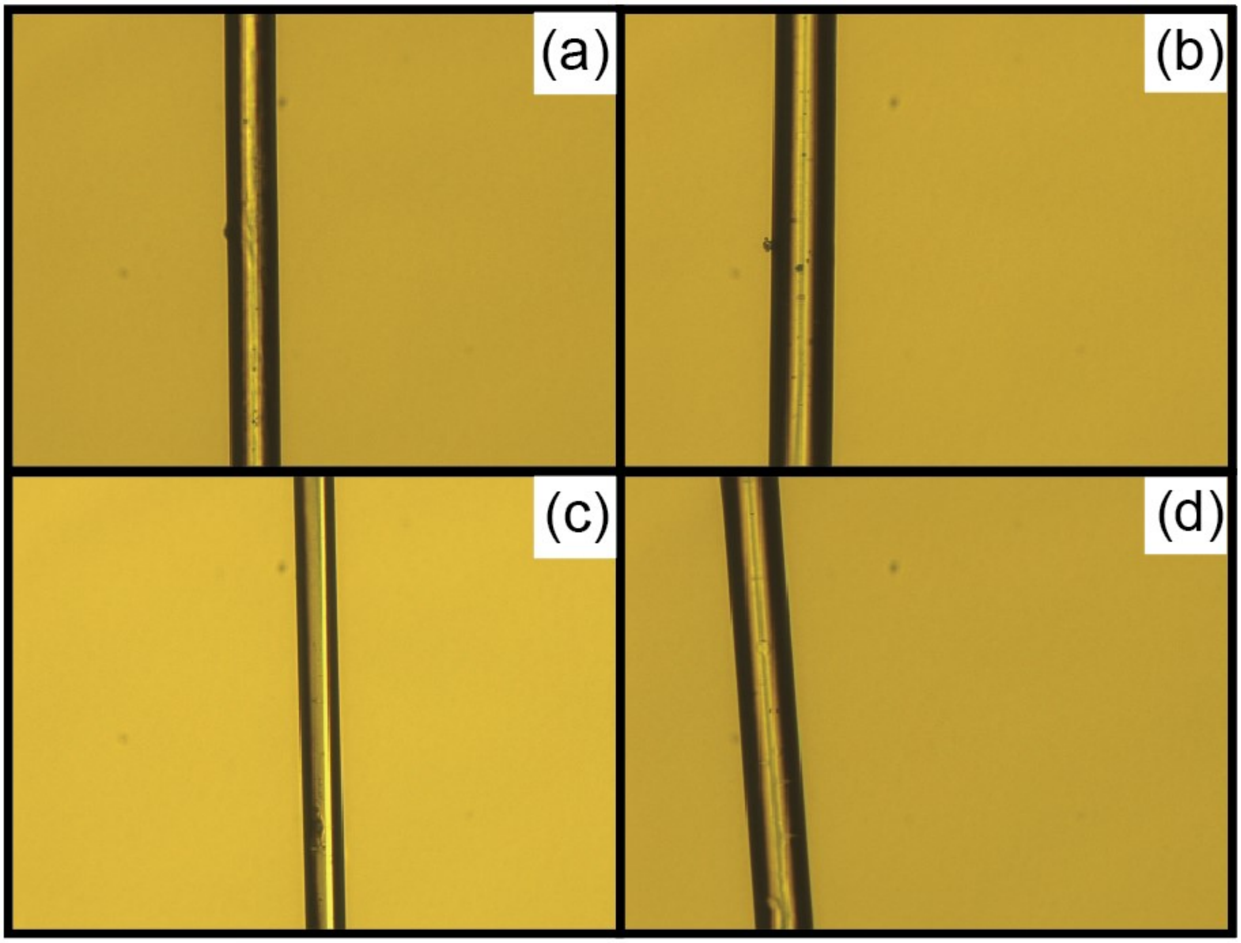

Fig. S7 Photographs of PAN/ILs fibers: (a) PAN/[C_2mim]Cl, (b) PAN/[C_2mim]Br, (c) PAN/[C_2mim]DCA, (d) PAN/[C_2mim]TCM.

Reference

1. H. J. Martin, H. Luo, H. Chen, C.-L. Do-Thanh, L. T. Kearney, R. Mayes, A. K. Naskar and S. Dai, *ACS Applied Materials & Interfaces*, 2020, 12, 8663-8673.
